# Heterozygous loss of function of *NR4A2* is associated with intellectual deficiency, rolandic epilepsy, and language impairment

**DOI:** 10.1002/ccr3.2260

**Published:** 2019-07-11

**Authors:** Luiza L. P. Ramos, Fabiola P. Monteiro, Leticia P. B. Sampaio, Larissa A. Costa, Mara D. O. Ribeiro, Erika L. Freitas, Joao P. Kitajima, Fernando Kok

**Affiliations:** ^1^ Mendelics Genomic Analysis Sao Paulo Brazil; ^2^ Department of Neurology University of Sao Paulo School of Medicine Sao Paulo Brazil

**Keywords:** intellectual deficiency, *NR4A2*, rolandic epilepsy

## Abstract

Recognition of a de novo mutation in *NR4A2* associated with a neurodevelopmental phenotype reinforces its role in 2q23q24 microdeletion syndrome. Using the proband WES data and the probability of loss‐of‐function intolerance index (pLi) set at 1.0 (highest intolerance constraint), we could target *NR4A2* as the candidate gene in this patient.

## 1 BACKGROUND

*NR4A2* haploinsufficiency due to whole gene deletion was recently associated with neurodevelopmental and language delay phenotypes. We report a heterozygous loss‐of‐function variant in *NR4A2* [nuclear receptor subfamily 4 group A2, OMIM *601828] in a patient with epilepsy, language impairment, and intellectual deficiency. This finding confirms that *NR4A2* is responsible for the features associated with the 2q23q24 microdeletion syndrome.

*NR4A2* haploinsufficiency due to whole gene deletion was recently associated with neurodevelopmental and language delay phenotypes by Reuter et al^1^ and Lévy et al.^2^ This finding strongly suggests that *NR4A2* is the critical gene for 2q24.1 microdeletion syndrome.

## 2 CASE PRESENTATION

Herein, we report a heterozygous loss‐of‐function variant in *NR4A2* [nuclear receptor subfamily 4 group A2, OMIM *601828] in a patient with epilepsy, language impairment, and intellectual deficiency. The individual is the first son of nonconsanguineous Caucasian parents, who was born after an uneventful pregnancy and labor. During infancy, he presented poor feeding and gastrointestinal symptoms such as gagging due regurgitation and colic. The boy had normal motor development and growth parameters, but his speech development was delayed. Dysmorphic features were absent. At age of 5 years, he presented seizures during sleep. Electroencephalogram (EEG) background activity was normal; interictal activity was characterized by high amplitude bilateral sharp waves paroxysms in the centroparietotemporal region, with marked frequency increase during drowsiness and sleep. This pattern was suggestive of rolandic epilepsy but did not characterize electrical status epilepticus during slow‐wave sleep (ESESS). Sporadic motor focal seizures persisted for 2 years, usually during sleep, and are currently under control with an association with clobazam and sulthiame. His EEG did not change along these years. Brain MRI was normal. Formal cognitive testing at 9 years of age with WISC‐IV (Wechsler Intelligence Scale for Children) revealed results below −2 SD. Verbal comprehension and operational memory were particularly impaired, with a calculated IQ of 63 (1st percentile) and 52 (0.1th percentile), respectively. He currently presents learning disability and in inclusive education at school.

**Figure 1:**
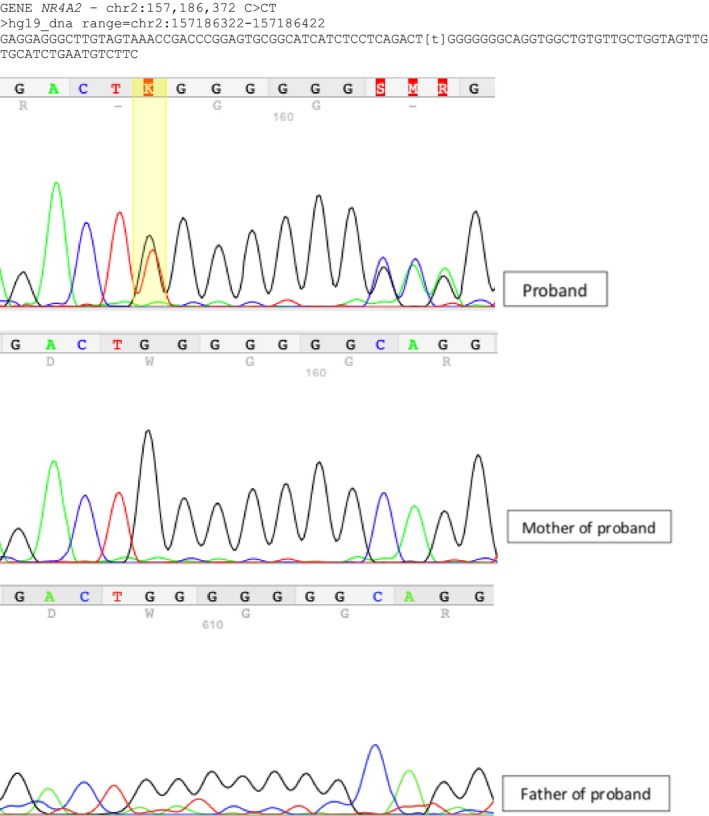
Electropherogram showing the presence of heterozygous c.326dupA variant in patient and its absence in parents, characterizing a de novo mutation

Whole exome sequencing revealed a frameshift variant in *NR4A2* (c.326_327insA or c.326dupA, ENST00000339562) which is predicted to cause a premature stop codon [p.(Ser110Valfs*2)] leading to either a nonsense‐mediated decay or a shortened nonfunctional protein. Sanger sequencing confirmed its presence in the patient, and additional study of the parents showed that it occurred as a de novo event Figure 1. No other potentially pathogenic variants in genes previously associated with intellectual deficiency or rolandic epilepsy, such as *GRIN2A* and *KCNB1*, were found.

## 3 DISCUSSION

To our knowledge, this is the first time a point mutation leading to loss of function in *NR4A2* is reported. Our patient's phenotype is similar to the previously described in individuals with de novo microdeletions encompassing *NR4A2* at the 2q24.1 region and whose clinical manifestations were mainly characterized by language and cognitive delay.^1^, ^2^, ^3^, ^4^, ^5^, ^6^ Our patient also presented focal motor seizures in the rolandic epilepsy spectrum, which expands the clinical manifestations related to *NR4A2* haploinsufficiency and reinforces the predominance of language impairment associated with this condition.

Barge‐Schaapveld et al^3^ and Shimojima et al^6^ suggested previously that *GPD2* haploinsufficiency was the most likely to be responsible for the 2q23q24 microdeletion phenotype. However, *GPD2* probability of loss‐of‐function intolerance (pLi) is zero and over 100 loss‐of‐function variants are reported in ExAC,on the other hand, *NR4A2* has a pLi = 1 and no loss‐of‐function variants in ExAC.^7^ This finding reinforces the probability of *NR4A2* being the main gene responsible for the phenotype of 2q23q24 microdeletion. It also demonstrates the utility of pLi to funnel down candidate genes for a microdeletion phenotype.

Seizures pattern and EEG of the patient are within the spectrum of rolandic epilepsy, in which language and behavior abnormalities are also common. In the more severe end of this continuum, Landau‐Kleffner syndrome and ESESS, developmental delay, language regression, and behavioral disorders are commonly observed.^8^ Pathogenic variants in other genes, such as *GRIN2A* and *KCNB1*, have been previously reported associated with rolandic epilepsy spectrum^9^, ^10^ reinforcing this neurocognitive endophenotype.

Our finding confirms that *NR4A2* is responsible for most, if not all, clinical features associated with the 2q23q24 microdeletion syndrome, furthermore, reinforces its implication in language impairment, and adds epilepsy to the phenotype.

## CONFLICT OF INTEREST

None declared.

## AUTHOR CONTRIBUTIONS

LLPR: was responsible for data collection and manuscript writing. FPM, LAC, and MDOR: were involved in whole genome sequencing analysis and contributed in manuscript writing. LPBS: performed epilepsy investigation. ELF: is the laboratory senior scientist and was responsible for NGS and Sanger sequencing. JPK: is the senior bioinformatician and was involved in variants filtering and prioritization. FK: was responsible for patient's care, genetic analysis and phenotype characterization as well as manuscript edition.
